# Knowledge of medical professionals, their practices, and their attitudes toward traditional Chinese medicine for the prevention and treatment of coronavirus disease 2019: A survey in Sichuan, China

**DOI:** 10.1371/journal.pone.0234855

**Published:** 2021-03-16

**Authors:** Jing Pu, He Mei, Li Lei, Daiwen Li, Jiekun Zhao, Bin Li, Haiyan Wang, Ying Ma, Xiao Bo Du

**Affiliations:** 1 Department of Traditional Chinese Medicine, Mianyang Central Hospital, Mianyang, Sichuan, China; 2 Department of Nursing, Mianyang Central Hospital, Mianyang, Sichuan, China; 3 Scientific Research and Education Department, Mianyang Central Hospital, Mianyang, Sichuan, China; 4 Department of Oncology, Mianyang Central Hospital, Mianyang, Sichuan, China; Chinese Academy of Medical Sciences and Peking Union Medical College, CHINA

## Abstract

This study aimed to investigate the knowledge, practices, and attitudes of medical professionals toward Traditional Chinese Medicine (TCM) for the prevention and treatment of coronavirus disease 2019 (COVID-19). All 401 medical professionals were surveyed using an anonymous with an investigator using the Questionnaire star APP. The participants answered 14 questions; of the 401 participants, 55.2% agreed with the statement “TCM can be used for the prevention and treatment of COVID-19,” 40.4% remained neutral, and 4.4% disagreed. Moreover, 75.3% agreed with the statement “There is no specific drug for COVID-19,” 67% agreed with the statement “TCM can develop immunity to COVID-19” and 62.1% agreed with “TCM can alleviate the symptoms of patients with COVID-19.” Meanwhile, 69.1% were aware that TCM has been recommended for COVID-19 by the National Health Commission of the People’s Republic of China. Regarding the selection of sources of knowledge on whether “TCM can be used for the prevention and treatment of COVID-19,” There were 277, 123, 82, 369, and 17 participants selected sources from “Hospital training,” “Academic journals,” “Academic Conferences,” “Social media platforms (such as WeChat)” and “Others,” respectively. Further, 358 participants will take TCM for the prevention of COVID-19. Multiple logistic regression analysis showed that age, major and received TCM treatment within the last five years were independent factors affecting the participants’ attitudes. In the absence of specific drugs for COVID-19, more than half of the participants agreed that TCM could be used for the prevention and treatment of COVID-19 and most participants are willing to take TCM to prevent COVID-19, although unsure about its effectiveness. The main information sources on TCM for the treatment and prevention of COVID-19 were social platforms and hospital training.

## Introduction

In December 2019, coronavirus disease 2019 (COVID-19) was discovered in Wuhan, later spreading throughout China [1, 2]. The complications of COVID-19 included acute respiratory distress syndrome, RNAaemia, acute cardiac injury, and secondary infections. Thirty-two percent of the patients were admitted to an intensive care unit and 15% of them died [3]. The treatment of COVID-19 remains unclear and no antiviral treatment has been proven to be effective [4]. Preclinical evidence demonstrated the potent efficacy of remdesivir to treat severe acute respiratory syndrome coronavirus and Middle East respiratory syndrome coronavirus infections [5, 6]. Thus, a randomized controlled trial has been quickly initiated to assess the efficacy and safety of the combined use of lopinavir and ritonavir in patients hospitalized with COVID-19. Although corticosteroids were used frequently for the treatment of severely ill patients, there is no evidence that systematic corticosteroid treatment is beneficial or harmful for patients infected with COVID-19.

Although there are no effective therapeutic drugs for COVID-19, the National Health Commission of the people’s Republic of China recommended, in the Guideline of Diagnosis and Treatment Plan for COVID-19 (5^th^ edition), that lopinavir and ritonavir can be used [7], and that Traditional Chinese Medicine (TCM) can also be used for different phases of COVID-19. However, there is no evidence to prove that TCM is beneficial for patients with COVID-19. Whether TCM can be used for the prevention and treatment of COVID-19 remains unclear, and the attitudes of medical professionals with regard to this need are to be examined as well.

The purpose of this study was to investigate the knowledge of Chinese medical professionals, their practices, and their attitudes toward TCM for the prevention and treatment of COVID-19 and then assess factors influencing their decisions.

## Methods

### Subjects

This study was conducted between February 7 and 10, 2020. All 401 medical professionals who worked in the 10 hospitals in Sichuan province were surveyed using an anonymous questionnaire that was sent from an investigator using the Questionnaire star APP, a professional online questionnaire survey, evaluation, and voting platform. This study was approved by the Medical Ethics Committee of Mianyang Central Hospital (No: P2020005). Because the medical professionals completed the survey questionnaire anonymously, and data were also analyzed anonymously, informed consent was not obtained from the participants.

### Questionnaire design and data collection

Based on a previous survey, which was used to investigate the attitudes of radiation oncologists toward percutaneous endoscopic gastrostomy in patients with head and neck cancer and eating difficulties [8], a self-reported questionnaire was developed by one of our investigators. When the preliminary design of our questionnaire was completed, it was reviewed by two different parties. The first group included those that were familiar with our topic, and who could evaluate whether our questions successfully captured the topic. The second review was conducted by a question construction expert, who ensured that the survey did not contain common errors such as leading, confusing, or double-barreled questions. Then, we conducted a pilot test with a sample of 20 medical professionals (doctors and nurses). Afterwards, we collected the responses into a spreadsheet to clean the data, performed the principal components analysis, and checked for internal consistency. According to the results of the principal components and internal consistency, we modified the questionnaire and performed another pilot test and analyzed the data again. The Kaiser-Meyer-Olkin Measure of Sampling Adequacy was 0.658, the p value of the Bartlett Test of Sphericity was <0.003 and Cronbach’s Alpha was 0.726, and based on these validations, we finally started our formal survey. The questionnaire was in Chinese and comprised four parts (S1 Questionnaire) with 14 questions. First, the participants were asked to provide their demographic data (e.g., gender and age). The were also asked whether they had received TCM treatment in the recent 5 years. Second, the participants were asked about their attitudes toward TCM for the prevention and treatment of COVID-19. The answers were recorded using a five-point Likert scale that ranged from “very much agree” to “very much disagree.” Third, the participants were asked about their awareness of COVID-19 treatment with TCM. Fourth, the participants were asked whether they will take TCM for the prevention of COVID-19. All questionnaires were exported and used in the analysis (100% response rate).

### Statistical analyses

Results were analyzed statistically using SPSS 22.0. Standard descriptive statistics were reported, the χ2-test was used to assess associations between demographic variables and attitudes of Chinese medical professionals toward TCM for the prevention and treatment of COVID-19. Multiple logistic regression was carried out to examine whether attitudes correlated with other factors. A two-sided analysis was used and a p-value <0.05 was considered to indicate statistical significance.

## Results

### General characteristics of participants

Table 1 shows the characteristics of the participants. The 401 participants consisted of 161 doctors and 240 nurses.

**Table 1:** Demographic characteristics of participants.

characteristic	No. of Participants (N = 401)	%
Sex		
Male	85	21.2
female	316	78.8
Age(years)		
21–30	174	43.4
31–40	107	26.7
41–50	91	22.7
≥51	29	7.2
Professional level		
Primary	186	46.4
Middle	122	30.4
Advanced	93	23.2
Occupation		
Doctor	161	40.1
Nurse	240	59.9
Time since qualification (years)		
1–5	121	30.2
6–10	116	28.9
11–15	34	8.5
16–20	30	7.5
≥21	100	24.9
Major		
Chinese medicine	233	58.1
West medicine	168	41.9
Received TCM treatment in five years		
Yes	238	59.4
No	163	40.6

### Knowledge, practices, and attitudes toward TCM for the prevention and treatment of COVID-19

The knowledge of the participants, their practices, and attitudes toward TCM for the prevention and treatment of COVID-19 are shown in Table 2, and we performed a subgroup analysis to understand the impact of occupations on the survey. For the statement “Attitudes toward TCM for prevention and treatment of COVID-19,” a larger percentage of doctors opted for the “very much agreed” (31.1% vs. 15.4%) option. In the case of the statement, “There is no specific drug for COVID-19,” more doctors selected the “very much agreed” (54.7% vs. 35%) option, while more nurses selected the “Neutral” (28.8% vs. 9.9%) option. Furthermore, in the response to the query “TCM can develop immunity to COVID-19,” more doctors opted for the “very much agreed” (31.1% vs. 18.3%) option, while more nurses selected the “Neutral” (32.9% vs. 22.4%) option. Further, in response to the statement “TCM can alleviate the symptoms of COVID-19 patients,” more doctors selected the “very much agreed” (34.2% vs. 17.1%) option, whereas more nurses selected the “Neutral” (44.4% vs. 25.5%) option. Moreover, it must be emphasized that a higher number of doctors were aware that TCM had been recommended for COVID-19 (85.1% vs. 58.3%). Furthermore, there was no difference in the response between the doctors and the nurses for the statement “Eat TCM for prevention of COVID-19, which is provided by their hospital.”

**Table 2:** Knowledge, practices and attitudes toward TCM for prevention and treatment of COVID-19.

	Participants	Doctor	Nurse	p (doctor VS nurse)
	N(%)	N(%)	N(%)	
Attitudes toward TCM for prevention and treatment of COVID-19				0.001
very much agree	87(21.7)	50(31.1)	37(15.4)	
Somewhat agree	134(33.5)	47(29.2)	87(36.3)	
Neutral	162(40.4)	55(34.2)	107(44.5)	
Somewhat disagree	13(3.2)	8(5.0)	5(2.1)	
very much disagree	5(1.2)	1(0.6)	4(1.7)	
There is no specific drug for COVID-19				0.001
very much agree	172(42.9)	88(54.7)	84(35.0)	
Somewhat agree	130(32.5)	51(31.7)	79(32.9)	
Neutral	85(21.2)	16(9.9)	69(28.8)	
Somewhat disagree	9(2.2)	2(1.2)	7(2.9)	
very much disagree	5(1.2)	4(2.5)	1(0.4)	
The TCM can develop immunity from COVID-19				0.008
very much agree	94(23.4)	50(31.1)	44(18.3)	
Somewhat agree	175(43.6)	66(41.0)	109(45.4)	
Neutral	115(28.8)	36(22.4)	79(32.9)	
Somewhat disagree	13(3.2)	8(5.0)	5(2.1)	
very much disagree	4(1.0)	1(0.5)	3(1.3)	
TCM can alleviate the symptoms of COVID-19 patients				0.001
very much agree	96(23.9)	55(34.2)	41(17.1)	
Somewhat agree	153(38.2)	60(37.3)	93(38.8)	
Neutral	138(34.5)	41(25.5)	97(40.4)	
Somewhat disagree	9(2.2)	4(2.5)	5(2.1)	
very much disagree	5(1.2)	1(0.5)	4(1.6)	
Knew that TCM bad been recommended for COVID-19				0.001
Yes	277(69.1)	137(85.1)	140(58.3)	
No	124(30.9)	24(14.9)	100(41.7)	
Eat TCM for prevention of COVID-19, which provide by their hospital				0.282
Yes	358(89.3)	147(91.3)	211(87.9)	
No	43(10.7)	14(8.7)	29(12.1)	

### Attitudes toward TCM for the prevention and treatment of COVID-19

Up to 55.2% of the participants agreed with the statement “TCM can be used for the prevention and treatment of COVID-19,” 40.4% responded neutrally, and 4.4% disagreed with the statement.

### Knowledge of COVID-19 treatment with TCM

With regard to the source of information on “TCM used for the prevention and treatment of COVID-19,” There were 100, 184, 68, 47, and 2 participants selected single, two, three, four, and five sources, respectively. Further, there were 277,123, 82, 369, and 17 participants stated that their sources came from “Hospital training,” “Academic journals,” “Academic Conferences,” “Social media platforms (such as WeChat)” and “Others,” respectively.

### Practices on prevention of COVID-19 with TCM

Three-hundred-and-fifty-eight participants will take TCM for the prevention of COVID-19, and 62.8% of them considered it unharmful and worth a try. Forty-three participants were unwilling to take TCM, 53.5% of them considered it ineffective, and 46.5% considered it harmful.

### Factors affecting participants’ attitudes

Sex, age, major, occupation and received TCM treatment in the last five years affected participants’ attitudes toward TCM for the prevention and treatment of COVID-19 (Table 3). Multiple logistic regression analysis showed that age, major and received TCM treatment in the last five years were the independent factors affecting participants’ attitudes (Table 4).

**Table 3:** Analyses of the related factors affecting participants’ attitudes.

	Very much Agree	Somewhat agree	Neutral	Somewhat disagree	Very much disagree	p
	N(%)	N(%)	N(%)	N(%)	N(%)	
Sex						0.004
Male	30(35.3)	21(24.7)	28(32.9)	5(5.9)	1(1.2)	
Female	57(18.0)	113(35.8)	134(42.4)	8(2.5)	4(1.3)	
Age(years)						0.004
21–30	23(13.2)	60(34.5)	81(46.6)	6(3.4)	4(2.3)	
31–40	34(31.8)	27(25.2)	43(40.2)	2(1.9)	4(0.9)	
41–50	19(20.9)	35(38.5)	32(35.2)	5(5.5)	0(0.0)	
≥51	11(37.9)	12(41.4)	6(20.7)	0(0.0)	0(0.0)	
Professional level						0.190
Primary	30(16.1)	60(33.3)	86(46.2)	5(3.2)	2(1.1)	
Middle	32(26.2)	42(34.4)	42(34.4)	3(2.5)	3(2.5)	
Advanced	25(126.9)	30(32.3)	34(36.6)	4(4.3)	0(0.0)	
Occupation						0.001
Doctor	50(31.1)	47(29.2)	55(34.2)	8(5.0)	1(0.6)	
Nurse	37(15.4)	87(36.3)	107(44.6)	5(2.1)	4(1.7)	
Time since qualification						0.205
1–5	18(14.9)	43(35.5)	54(44.6)	5(4.1)	1(0.8)	
6–10	28(24.1)	33(28.4)	49(42.2)	2(1.7)	4(3.4)	
11–15	10(29.4)	7(20.6)	16(47.1)	1(2.9)	0(0.0)	
16–20	7(23.3)	10(33.3)	11(36.7)	2(6.7)	1(0.6)	
≥21	24(24.0)	41(41.0)	32(32.0)	3(3.0)	0(0.0)	
Major						0.001
West medicine	16(6.9)	80(34.3)	122(52.4)	11(4.7)	4(1.7)	
TCM	71 (42.3)	54(32.1)	40(23.8)	2(1.2)	1(0.6)	
Received TCM treatment						0.001
Yes	76(31.9)	84(35.3)	69(29.0)	6(2.5)	3(1.3)	
No	11(6.7)	50(30.7)	93(57.1)	7(4.3)	2 (1.2)	

**Table 4:** Multiple logistic regression was used to analyze the independent factors affecting participants’ attitudes.

	χ2	Degree of freedom	p
Sex	3.7	4	0.442
Age	34.5	12	0.001
Occupation	2.9	4	0.577
Major	68.4	4	0.001
Received TCM treatment	25.5	4	0.001

## Discussion

At the time the survey was conducted no clinical study had indicated that there is a specific drug for the treatment of COVID-19. Cao et al. reported there were no differences in mortality among patients with COVID-19 who did or did not receive antimicrobial [9, 10]. Wang et al. reported that remdesivir was not associated with significant clinical benefits for severe COVID-19 [11]. Therefore, more than 70% participants agreed with the statement “There is no specific drug for COVID-19.” So far, there is no strong evidence supporting TCM use for the prevention and treatment of COVID-19 [12]. However, 55.2% of participants agreed with the statement “TCM can be used for the prevention and treatment of COVID-19,” and up to 89.3% of the participants were willing to consider TCM to prevent COVID-19. Third, more than 60% of the participants agreed that TCM can improve immune function and alleviate symptoms in patients. Fourth, 56.2% participants thought that TCM is unharmful and should be worked with. The results of this survey are consistent with those of the previous survey on nursing students’ attitudes toward TCM [13]. Forty-five percent of 263 Chinese nursing students reported a positive attitude towards TCM use, 52% were neutral, and only 3% were negative. In another study, the attitude towards TCM was positive in 41%, neutral in 52% and negative in only 6% [14].

Subgroup analysis showed that there was significant difference in the knowledge and attitudes between doctors and nurses. The difference in attitudes between the doctors and nurses toward TCM could be majorly attributed to the following: First, doctors and nurses had different concerns while treating and attending to patients. Therefore, despite their different attitudes towards TCM, doctors and nurses reach a consensus while discharging their duties toward their patients. Our result show that nearly 90% medical professionals willing to eat TCM for the prevention of COVID-19. Major and age and received TCM treatment were the factors affecting participants’ attitudes. Older participants tended to agree more with “TCM can be for the prevention and treatment of COVID-19,” with 79.3% of participants selecting “very much agree and somewhat agree” in the age group of >51 years, whereas only 47.7% in the age group between 21 and 30 years did. The same result was seen in a survey of attitudes toward TCM among Western-trained doctors. The reason for these results could be that older doctors have more training in TCM than younger doctors [15]. Participants with major of TCM tended to agree more with “TCM can be for the prevention and treatment of COVID-19,” with 74.4% of participants selecting “very much agree and somewhat agree” in TCM group, whereas only 41.2% in West Medicine group did. The results can be easily explained: participants’ responses were dictated by their experience. There were 59.4% of participants who had received TCM treatment, consistent with the previous survey on the application of TCM in residents of Beijing [16], and 56.8% (1937/3410) of residents had used herbal decoction. Among the participants who had received TCM treatment, 67.2% of them selected “very much agree and somewhat agree;” however, only 37.4% of the participants had not received TCM treatment. This was because they believed in the effectiveness of TCM and had received TCM treatment in the past, it is reasonable that they selected “very much agree and somewhat agree.”

Concerning the information sources on “TCM used for the prevention and treatment of COVID-19,” 75% of participants selected more than two sources. The first source selected was social media platforms (such as WeChat), which have been used in different fields, including medicine [17–19]. In China, many medical subscription accounts and official accounts can be found on social media platforms. As information on social media platforms is timely and easily accessible [20, 21], it has become the main channel for medical professionals [22, 23]. After February 20, all academic meetings had been canceled and social media platforms became the first choice for obtaining professional knowledge [24]. As many hospitals have executed all-employee training on the Guideline of Diagnosis and Treatment Plan for COVID-19 (5th), the second source is “Hospital training.” There is a need for more training on TCM prevention and treatment of COVID-19 for medical professionals because of all the employees, only 69.1% of the participants knew that TCM was recommended for use in the treatment of different phases of COVID-19 by the National Health Commission of the People’s Republic of China. The reason is that few participants participated took part in this study, as they focused on the prevention and triage while ignoring treatment which is one limitation of this study. Studies on COVID-19 were few and academic conferences had been canceled. Thus, very little information could be obtained from “Academic journals” and “Academic Conferences.”

Up to 89.3% of the participants were willing to take TCM to prevent COVID-19. However, out of them, 62.8% thought that TCM was unharmful and should be tried, although they were not sure about its effectiveness. This result is in line with the current situation; there is no evidence to show that TCM is effective in the treatment of COVID-19 [12]. To determine the efficacy in TCM for the treatment and prevention of COVID-19, it is necessary to provide sufficient evidence from clinical trials. In all participants, only 33.16% thought that TCM was effective, lower than the results of a previous survey of Beijing residents [16] in which 80.7% (2753/3410) of the residents believed that TCM herbal therapy was effective for disease treatment. The reason could be that the medical professionals surveyed in the current study had professional knowledge, causing them to make objective judgments. Only 5% of participants thought that TCM was harmful, and only 4.4% of participants who disagreed that TCM could be used for the prevention and treatment of COVID-19.

The limitations of this study are as follows: First, whether or not they participated in the treatment of COVID-19 patients was not designed as a question in the survey, which may have affected their response. Second, the TCM includes Chinese herbal medicine, Chinese patent medicine, Traditional Chinese medicine injection, acupuncture, and other treatments, but we did not specify these types in our questionnaire, which may have confused the participants, thereby affecting their response. Third, we only performed a subgroup analysis to understand the impact of occupations (doctors and the nurses) on the survey, we did not evaluate the impact of varied specialties (Chinese medicine and West medicine) of the doctors or nurses.

## Conclusion

In the absence of specific drug for COVID-19, over half of the participants agreed that TCM can be used for the prevention and treatment of COVID-19 and most participants were willing to take TCM to prevent COVID-19, although unsure about its effectiveness. The main information sources on TCM for the treatment and prevention of COVID-19 were social media platforms and hospital training.

## Supporting information

S1 Questionnaire Survey on medical professionals’ attitudes toward traditional Chinese medicine for prevention and treatment of corona virus disease 2019 (COVID-19). (DOCX)

S1 Raw data (XLSX)

S2 Raw data (DOCX)
