# Supplementary material for: Knowledge of medical professionals, their practices, and their attitudes toward traditional Chinese medicine for the prevention and treatment of coronavirus disease 2019: A survey in Sichuan, China
Source: PLoS One. 2021 Mar 16;16(3):e0234855. doi: 10.1371/journal.pone.0234855 (PMC7963037; doi:10.1371/journal.pone.0234855)
Supplement: S1 Questionnaire — (DOCX) [file pone.0234855.s001.docx]

**Survey On Medical Professionals ' Attitudes Toward Traditional Chinese Medicine For Prevention And Treatment Of Corona Virus Disease 2019( COVID-19)**

This form is only used for scientific research and your information will remain completely confidential. Please provide the most relevant options in the list. Any other answers you may wish to add can be also listed separately.

Thank you for your support.

1. Gender (single choice )

○ male ○ female

2. Your age group (single choice )

○ 21-30 years ○ 31-40 years ○ 41-50 years ○ 51 years and above

3. Professional level (single choice )

○ primary level ○ Middle○ Advanced

4. Your occupation category (single choice )

○ doctor ○ nurse

5. Your working years (single choice )

○ 1-5 years ○ 6-10 years ○ 11-15 years ○ 16-20 years ○ 21 years and above

6. Your major (single choice )

○ traditional Chinese medicine ○ western medicine

7. Have you received TCM treatment in recent 5 years? (single choice )

○ no ○ yes

8, What's your opinion on the prevention and treatment of traditional Chinese medicine for COVID-19?(single choice )

○ very agree ○ relatively agree ○ neutral ○ relatively disagree ○ very disagree

9. There is no specific drug for COVID-19. (single choice )

○ very agree ○ relatively agree ○ neutral ○ relatively disagree ○ very disagree

10. Do you think that Chinese medicine can develop immunity from COVID-19 (single choice )

○ very agree ○ relatively agree ○ neutral ○ relatively disagree ○ very disagree

11. Do you think TCM can alleviate the symptoms of COVID-19 patients? (single choice )

○ very agree ○ relatively agree ○ neutral ○ relatively disagree ○ very disagree

12. Do you knew that TCM have been recommended to use for different phase of COVID-19 by National Health Commission of the People’s Republic of China. (single choice )

○ know ○ don't know

13 The knowledge sources of "TCM used for prevention and treatment of COVID-19" (multiple choice)

○ hospital training ○ academic journals ○ academic conferences ○ social platforms (such as Wechat) ○ others

14, Do you eat the TCM for prevention of COVID-19 , which provide by you hospital. (single choice )

○ Yes (reason: ○ because it's effective ○ try it, it's OK anyway)

○ no (reason: ○ invalid ○ possibly harmful)
