# Supplementary material for: Knowledge of medical professionals, their practices, and their attitudes toward traditional Chinese medicine for the prevention and treatment of coronavirus disease 2019: A survey in Sichuan, China
Source: PLoS One. 2021 Mar 16;16(3):e0234855. doi: 10.1371/journal.pone.0234855 (PMC7963037; doi:10.1371/journal.pone.0234855)
Supplement: S2 Raw data — (DOCX) [file pone.0234855.s003.docx]

医学专业人员对中医预防和治疗冠状病毒肺炎（COVID-19）的态度调查

此表格仅用于科学研究，您的信息将完全保密。请在列表中提供最相关的选项。谢谢你的支持。您可能希望添加的任何其他答案也可以单独列出。

1性别（单选）

○男○女

2你的年龄组（单选）

○21-30岁○31-40岁○41-50岁○51岁及以上

三。专业水平（单选）

○初级○中级○高级

4您的职业类别（单选）

○医生○护士

5你的工作年限（单选）

○1-5年○6-10年○11-15年○16-20年○21年及以上

6你的专业（单选）

○中医○西医

7你近5年来接受过中医治疗吗？（单选）

○否○是

8，您对COVID-19中药防治的看法如何？（单选）

○非常同意○相对同意○中立○相对不同意○非常不同意

9COVID-19没有特定的药物。（单选）

○非常同意○相对同意○中立○相对不同意○非常不同意

10你认为中药可以增强COVID-19患者（单选）的免疫力吗

○非常同意○相对同意○中立○相对不同意○非常不同意

11你认为中医能缓解COVID-19患者的症状吗？（单选）

○非常同意○相对同意○中立○相对不同意○非常不同意

12您是否知道中华人民共和国国家卫生委员会建议TCM用于COVID-19的不同阶段的治疗。（单选）

○知道○不

13“中医药用于预防和治疗COVID-19”（多选）的知识来源

○医院培训○学术期刊○学术会议○社交平台（如微信）○其他

14，您是否吃了由您医院提供的预防COVID-19的中药。（单选）

○是（原因：○因为它有效○尝试它，无论如何都好）

○否（原因：○无效○可能有害）
